# Global mammographic asymmetry and short-term breast cancer risk by breast density: a nationwide screening cohort of 5.5 million women

**DOI:** 10.1016/j.breast.2026.104708

**Published:** 2026-01-21

**Authors:** Sangjun Lee, Soyeoun Kim

**Affiliations:** a Integrated Major in Innovative Medical Science, Seoul National University Graduate School, Seoul, Republic of Korea; b Department of Preventive Medicine, Seoul National University College of Medicine, Seoul, Republic of Korea; c Cancer Research Institute, Seoul National University, Seoul, Republic of Korea; d Biomedical Research Institute, Seoul National University Hospital, Seoul, Republic of Korea

**Keywords:** Breast cancer, Mammography, Breast asymmetry, Breast density, Screening, Cohort study

## Abstract

Global mammographic asymmetry (GA) is generally considered benign, and its association with subsequent breast cancer risk is unclear. We examined whether GA on screening mammography predicts short-term and long-term breast cancer and whether this varies by Breast Imaging Reporting and Data System (BI-RADS) breast density. In this retrospective cohort study using the Korean National Health Insurance Service screening programme, we included women aged ≥40 years who underwent screening mammography in 2009–2010 and had no prior breast cancer. GA and BI-RADS density were recorded on baseline mammograms; incident invasive breast cancer through December 31, 2019 was ascertained from insurance claims. Cox proportional hazards models estimated adjusted hazard ratios (aHRs) for breast cancer associated with GA overall and by BI-RADS density and follow-up interval (<1, 1–2 and ≥2 years), adjusting for demographic, reproductive and lifestyle factors. Among 5,475,113 women, GA was present in 4.0 %. Overall, GA was associated with a modestly increased breast cancer risk (aHR 1.15; 95 % CI 1.11–1.19), strongest within 1 year of screening (aHR 1.90; 95 % CI 1.70–2.12). In women with BI-RADS 1 breasts, GA doubled overall risk (aHR 2.03) and quadrupled short-term risk (<1 year: aHR 4.14), whereas in BI-RADS 4 breasts GA did not increase overall risk (aHR 0.94). GA is uncommon but identifies women at substantially elevated short-term breast cancer risk, particularly those with non-dense breasts, and has limited long-term prognostic value in extremely dense breasts. These findings support consideration of short-interval follow-up or supplemental imaging when GA is reported in non-dense breasts.

## Highlights

• Global breast asymmetry on screening mammography predicts short-term breast cancer risk.
• Breast cancer risk after asymmetry is highest within one year of screening.
• Asymmetry doubles cancer risk in women with mostly fatty breasts.
• Asymmetry adds little long-term risk information in extremely dense breasts.
• Short-interval follow-up may benefit women with asymmetry and low breast density.

## 1 Introduction

Screening mammography occasionally reveals an asymmetric area of fibroglandular density, reported in approximately 3 % of examinations [1]. Among these asymmetries, global asymmetry (GA) refers to a unilateral increase in fibroglandular tissue involving one or more quadrants of a breast and visible on both standard craniocaudal and mediolateral oblique views [2]. In the absence of an associated mass, architectural distortion, or suspicious calcifications, GA is generally considered benign, and further diagnostic evaluation is not routinely indicated [2].

Although GA detected at a single screening encounter is usually regarded as benign, its potential association with an increased subsequent risk of breast cancer remains unclear. Mammographic breast density is a well-established risk factor—women with extremely dense breasts have approximately a four-to six-fold higher risk of developing breast cancer than those with predominantly fatty breasts [3]—but whether GA provides predictive information beyond overall density is uncertain. It has been suggested that not only the absolute amount of dense tissue, but also its spatial distribution and asymmetry, may reflect a biological predisposition to malignancy [4,5]. Supporting this hypothesis, several studies have found that density asymmetry is a stronger short-term risk indicator than either age or mean density alone [6,7]. These findings raise the possibility that GA could represent a mammographically occult cancer that becomes clinically manifest shortly thereafter. However, to our knowledge, no large population-based cohort study with extended follow-up has investigated this association.

We therefore conducted a nationwide, population-based cohort study of more than five million Korean women to examine whether GA identified on screening mammography is associated with subsequent incident breast cancer. We further assessed whether this association differs by baseline breast density and time since screening (short-term vs. long-term risk).

## 2 Methods

### 2.1 Study settings and study population

This retrospective nationwide cohort study was approved by the Institutional Review Board of Seoul National University Hospital (E−2406-021-1540) with a waiver of informed consent. Data were obtained from South Korea's National Health Insurance Service (NHIS), a compulsory health insurance system covering the entire Korean population [8]. A customized dataset from the NHIS claims database included sociodemographic characteristics, mortality data, healthcare utilization, and national health screening results [9].

**Fig. 1:**
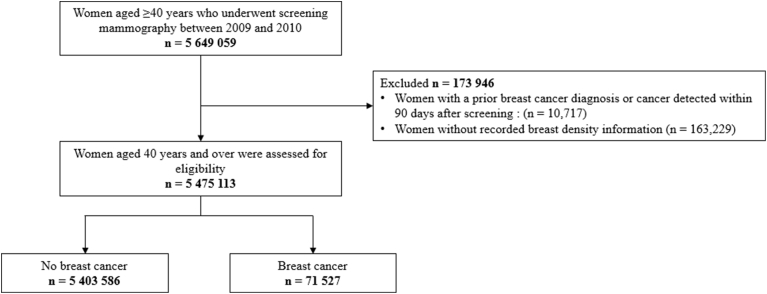
Flow diagram showing selection of study participants.

Women aged ≥40 years who underwent screening mammography between 2009 and 2010 were included (Fig. 1). Those with a prior breast cancer diagnosis or cancer detected within 90 days after screening (n = 10,717) and those without recorded breast density information (n = 163,229) were excluded. The final cohort comprised 5,475,113 women, who were followed from the baseline screening date until breast cancer diagnosis, death, or December 31, 2019.

**Fig. 2:**
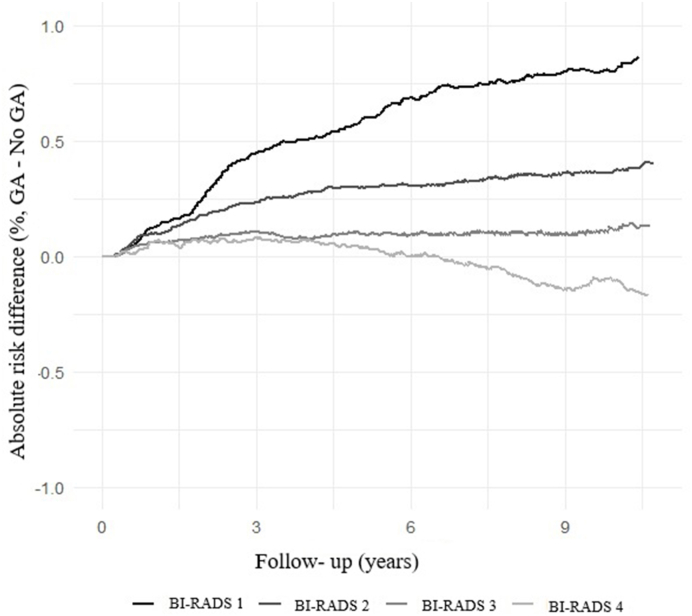
Absolute difference in cumulative breast cancer incidence (%) between women with and without GA over time, stratified by baseline BI-RADS breast density category.

### 2.2 Mammographic breast density and GA

Mammographic breast density and the presence of GA were recorded based on interpretations by certified radiologists at individual screening centers participating in the national screening program. Since 2009, the Korean National Breast Cancer Screening Program has utilized the fourth edition of the Breast Imaging Reporting and Data System (BI-RADS) lexicon to classify breast density into four standard categories: (a) BI-RADS density category 1, almost entirely fatty; (b) BI-RADS density category 2, scattered areas of fibroglandular density; (c) BI-RADS density category 3, heterogeneously dense; and (d) BI-RADS density category 4, extremely dense [10,11]. Radiologic assessment of mammograms included descriptors for notable findings such as masses, GA, architectural distortion, microcalcifications, and associated features. For this study, each participant's GA status was determined from the mammography report issued by the interpreting radiologist.

### 2.3 Development of breast cancer

The primary outcome was breast cancer incident, identified through insurance claims. Cases were defined using a combination of the International Classification of Diseases, 10th Revision diagnostic code for invasive breast cancer (C50) and a specialized “catastrophic illness” registration code for cancer. This claims-based case definition has demonstrated a sensitivity of 98.1 % when validated against the Korean Central Cancer Registry, which captures approximately 90 % of all cancer cases nationwide [12].

### 2.4 Confounder

Covariates for analysis included demographic, reproductive, and lifestyle factors collected at the time of breast cancer screening. Age at screening (years) was treated as a continuous variable. Menopausal status (premenopausal vs. postmenopausal), age at menarche (<15 vs. ≥15 years), and age at menopause (<52 vs. ≥52 years or still premenopausal) were recorded. Parity (nulliparous vs. parous) and history of breastfeeding (never vs. ever) were included, as were ever-use of oral contraceptives and ever-use of hormone replacement therapy (HRT, among postmenopausal women). Lifestyle factors included body mass index (BMI, <23 vs. ≥23 kg/m^2^, based on Asian BMI criteria) [13], smoking status (never vs. ever smoker), alcohol consumption (none vs. any), and regular physical activity (yes vs. no). Family history of breast cancer in a first-degree relative (yes vs. no) was also recorded. All covariates were self-reported, except BMI.

### 2.5 Statistical analysis

Baseline characteristics were compared between women with and without GA using chi-square tests for categorical variables and independent-samples t tests for continuous variables. For each stratum defined by GA status and BI-RADS density category, absolute risk of breast cancer over time was assessed by calculating cumulative incidence at 1, 5, and 10 years after baseline screening. We also calculated the incidence rate of breast cancer (per 100,000 person-years) using the number of breast cancer events and the total person-years of follow-up for each group To visualize differences in cumulative incidence between groups, we plotted absolute risk difference curves (cumulative incidence in the GA group minus that in the no-GA group) over the follow-up period, stratified by baseline BI-RADS density category.

We used Cox proportional hazards regression to estimate the association between baseline GA (yes vs. no) and subsequent breast cancer risk, expressed as hazard ratios (HRs) with 95 % confidence intervals (CIs). The primary model included GA as the exposure and was adjusted for age, BMI, age at menarche, menopausal status, age at menopause, parity, breastfeeding, oral contraceptive use, hormone replacement therapy, alcohol consumption, smoking, physical activity, and family history of breast cancer. To examine temporal variation in risk, analyses were stratified by follow-up interval—early (<1 year), intermediate (1–2 years), and late (≥2 years) after baseline screening—allowing estimation of separate HRs for each period. Participants who remained cancer-free were censored on December 31, 2019. Interaction between GA and BI-RADS density category was tested to determine whether associations differed by breast density. All tests were two-sided with P < .05. Analyses were performed using SAS version 9.4 (SAS Institute, Cary, NC) and R version 4.0.

## 3 Results

A total of 5,475,113 women met the inclusion criteria at baseline, of whom 218,329 (4.0 %) had GA. The median follow-up duration was 9.8 years. Table 1 showed the baseline characteristics of study participants. Women with GA were younger on average than those without GA (52.9 ± 9.5 vs. 55.2 ± 10.8 years; P < .001) and more often premenopausal (49.9 % vs. 42.4 %; P < .001). The distribution of BI-RADS density differed markedly by GA: BI-RADS 1 was less frequent in the GA group (10.1 % vs. 30.4 %), whereas BI-RADS 3 and 4 were more frequent (42.7 % vs. 28.2 % and 17.7 % vs. 14.5 %, respectively; P < .001). A slightly higher proportion of women with GA developed breast cancer during follow-up (1.7 % vs. 1.3 %; P < .001). Differences in several reproductive and lifestyle factors were small in magnitude, while parity and oral contraceptive use were similar between groups.

**Table 1:** Baseline characteristics of study participants according to the presence of global asymmetry on screening mammography.

	Global asymmetry				P-value
Characteristic	No		Yes		
	n = 5,256,784		n = 218,329		
**Age, years**	55.2 ± 10.8		52.9 ± 9.5		<0.001
**Breast cancer development**					
No	5,188,978	(98.7)	214,608	(98.3)	<0.001
Yes	67,806	(1.3)	3,721	(1.7)	
**BI-RADS density category**					
BI-RADS 1	1,600,151	(30.4)	22,093	(10.1)	<0.001
BI-RADS 2	1,414,079	(26.9)	64,220	(29.4)	
BI-RADS 3	1,482,327	(28.2)	93,264	(42.7)	
BI-RADS 4	760,227	(14.5)	38,752	(17.7)	
**Menopausal status**					
Premenopausal	2,229,796	(42.4)	108,950	(49.9)	<0.001
Postmenopausal	2,934,193	(55.8)	105,734	(48.4)	
Missing	92,795	(1.8)	3,645	(1.7)	
**Age at menarche, years**					
<15	1,173,392	(22.3)	54,453	(24.9)	<0.001
≥15	3,901,718	(74.2)	157,604	(72.2)	
Missing	181,674	(3.5)	6,272	(2.9)	
**Age at menopause, years**					
pre	2,229,796	(42.4)	108,950	(49.9)	<0.001
<52	1,788,854	(34.0)	60,212	(27.6)	
≥52	963,721	(18.3)	38,237	(17.5)	
Missing	274,413	(5.2)	10,930	(5.0)	
**Parity**					
Nulliparous	207,549	(3.9)	9,693	(4.4)	<0.001
Parous	4,958,617	(94.3)	205,033	(93.9)	
Missing	90,618	(1.7)	3,603	(1.7)	
**History of breastfeeding**					
Never	822,187	(15.6)	37,642	(17.2)	<0.001
Ever	4,434,597	(84.4)	180,687	(82.8)	
**Use of oral contraceptives**					
Never	4,278,300	(81.4)	177,000	(81.1)	<0.001
Ever	978,484	(18.6)	41,329	(18.9)	
**Use of hormone replacement therapy (postmenopausal women)**					
Never	2,482,079	(47.2)	86,212	(39.5)	<0.001
Ever	544,909	(10.4)	23,167	(10.6)	
Missing	2,229,796	(42.4)	108,950	(49.9)	
**BMI**					
<2 3	2,058,702	(39.2)	88,396	(40.5)	<0.001
≥23	2,840,851	(54.0)	115,001	(52.7)	
Missing	357,231	(6.8)	14,932	(6.8)	
**Smoking status**					
Never	4,659,704	(88.6)	192,639	(88.2)	<0.001
Ever	217,725	(4.1)	10,042	(4.6)	
Missing	379,355	(7.2)	15,648	(7.2)	
**Alcohol consumption**					
None	3,924,221	(74.7)	157,499	(72.1)	<0.001
Any	939,123	(17.9)	44,940	(20.6)	
Missing	393,440	(7.5)	15,890	(7.3)	
**Regular physical activity**					
No	1,416,117	(26.9)	53,597	(24.5)	<0.001
Yes	3,441,084	(65.5)	148,356	(68.0)	
Missing	399,583	(7.6)	16,376	(7.5)	
**Family history of breast cancer (first-degree relative)**					
No	5,177,991	(98.5)	214,564	(98.3)	<0.001
Yes	78,793	(1.5)	3,765	(1.7)	

Abbreviations: BMI, body mass index; BI-RADS, Breast Imaging Reporting and Data System.

Table 2 presents the absolute risks of breast cancer at 1, 5, and 10 years, along with incidence rates, stratified by GA status and BI-RADS density category. Among women without GA, breast cancer risk increased steadily with higher breast density. In contrast, among women with GA, absolute risks were higher than those without GA in BI-RADS categories 1–2, and similar in category 3 at 10 years. For example, among women with BI-RADS category 1 breasts, the presence of GA was associated with a 10-year risk of 1.53 %, roughly twice that of women with symmetric fatty breasts (0.72 %). In BI-RADS category 2, the 10-year risk was 1.53 % with GA versus 1.16 % without GA. In BI-RADS category 3, the 10-year risk was nearly identical between groups (approximately 1.71 % in both). Incidence rates broadly mirrored these differences—higher with GA in BI-RADS 1–3 and slightly lower in BI-RADS 4. Notably, in BI-RADS category 4, absolute risks were high for both groups and showed minimal difference—the 10-year risk was 2.03 % with GA versus 2.13 % without, corresponding to incidence rates of 202 versus 216 per 100,000 person-years, respectively.

**Table 2:** Absolute risk and incidence of breast cancer by global asymmetry and BI-RADS density category.

Global asymmetry	BI-RADS density category	Total no.	Total BC events	Person-years	1-year absolute risk (%)	5-year absolute risk (%)	10-year absolute risk (%)	BC incidence per 100,000 person-years
**No global asymmetry**								
	BI-RADS 1	1600151	11060	15339079.7	0.03	0.31	0.72	72
	BI-RADS 2	1414079	16012	13702838.9	0.06	0.51	1.16	117
	BI-RADS 3	1482327	24801	14393647.6	0.10	0.76	1.71	172
	BI-RADS 4	760227	15933	7365116.0	0.13	0.96	2.13	216
**Global asymmetry**								
	BI-RADS 1	22093	332	212178.9	0.15	0.88	1.53	156
	BI-RADS 2	64220	965	621695.4	0.16	0.81	1.53	155
	BI-RADS 3	93264	1663	904617.3	0.16	0.86	1.71	184
	BI-RADS 4	38752	761	376399.5	0.19	1.00	2.03	202

Abbreviations: BC, breast cancer; BI-RADS, Breast Imaging Reporting and Data System; No., number.

Fig. 2 illustrates the absolute difference in cumulative breast cancer incidence (%) between women with and without GA over time, stratified by baseline BI-RADS density category. In BI-RADS category 1, the cumulative 10-year incidence was approximately 1.53 % among women with GA versus 0.72 % among those without GA, representing an absolute difference of about 0.81 percentage points. BI-RADS category 2 showed a similar pattern, with higher incidence among women with GA (∼1.53 % vs. 1.16 % at 10 years; absolute difference, ∼0.37 percentage points). In BI-RADS category 3, the incidence curves for women with and without GA remained nearly superimposed throughout follow-up, with no appreciable difference at 10 years (∼1.71 % in both). In BI-RADS category 4, the curves were very similar early in the follow-up period; however, by 10 years, the cumulative incidence among women without GA slightly exceeded that among those with GA, corresponding to a small inverse difference in the extremely dense group (∼0.10 percentage points lower with GA; 2.03 % vs. 2.13 %).

Table 3 shows that, overall, the presence of GA was associated with a modest but statistically significant increase in breast cancer risk. Across the entire follow-up period, women with GA had approximately a 15 % higher hazard of developing breast cancer than those without GA (aHR, 1.15; 95 % CI, 1.11–1.19). This association was strongest shortly after screening and progressively attenuated over time. Within the first year after the baseline mammogram, women with GA had nearly double the risk of breast cancer compared with those without GA (aHR, 1.90; 95 % CI, 1.70–2.12). During the 1–2-year interval, the risk remained elevated but less pronounced (aHR, 1.38; 95 % CI, 1.24–1.53). Beyond 2 years, the difference in risk was modest but statistically significant (aHR, 1.07; 95 % CI, 1.03–1.11). Among women with BI-RADS category 1 breasts, the presence of GA was associated with a substantially higher short-term risk. Within the first year after screening, GA quadrupled the hazard of breast cancer (aHR, 4.14; 95 % CI, 2.91–5.88), and although the aHR decreased over time, it remained elevated throughout follow-up. In contrast, among women with BI-RADS category 4 breasts, GA did not confer any significant overall increase in risk (aHR, 0.94; 95 % CI, 0.87–1.01). During the first year after screening, asymmetry in BI-RADS category 4 breasts was associated with only a slight increase in hazard (aHR, 1.49; 95 % CI, 1.17–1.89), an effect far smaller than that observed in women with low-density breasts. By the 1–2-year interval, the aHR for GA in BI-RADS category 4 was 1.02 (95 % CI, 0.81–1.30).

**Table 3:** Breast cancer risk according to time since screening mammogram in relation to global asymmetry, stratified by BI-RADS density.

BI-RADS density category	Global asymmetry	Breast cancer risk										
		Total		<1 year			1–2 years			≥2 years		
		aHR	(95 % CIs)	aHR	(95 % CIs)		aHR	(95 % CIs)		aHR	(95 % CIs)	
Total												
	No	Ref.		Ref.			Ref.			Ref.		
	Yes	1.15	(1.11–1.19)	1.90	(1.70–2.12)		1.38	(1.24–1.53)		1.07	(1.03–1.11)	
BI-RADS 1												
	No	Ref.		Ref.			Ref.			Ref.		
	Yes	2.03	(1.82–2.26)	4.14	(2.91–5.88)		3.18	(2.35–4.31)		1.80	(1.59–2.04)	
BI-RADS 2												
	No	Ref.		Ref.			Ref.			Ref.		
	Yes	1.32	(1.24–1.41)	2.54	(2.07–3.12)		1.80	(1.49–2.18)		1.20	(1.11–1.29)	
BI-RADS 3												
	No	Ref.		Ref.			Ref.			Ref.		
	Yes	1.07	(1.02–1.12)	1.60	(1.35–1.90)		1.15	(0.98–1.35)		1.02	(0.97–1.08)	
BI-RADS 4												
	No	Ref.		Ref.			Ref.			Ref.		
	Yes	0.94	(0.87–1.01)	1.49	(1.17–1.89)		1.02	(0.81–1.30)		0.89	(0.82–0.97)	

Abbreviations: aHR, adjusted hazard ratio; CI, confidence interval; BI-RADS, Breast Imaging Reporting and Data System; Ref, reference.

All aHRs were adjusted for age, body mass index, age at menarche, menopausal status, age at menopause, parity, breastfeeding history, oral contraceptive use, hormone replacement therapy, alcohol consumption, smoking status, physical activity, and family history of breast cancer.

Table 4 presents breast cancer risk jointly by BI-RADS breast density and GA status across follow-up intervals, using BI-RADS category 4 without GA as the reference within each interval. Within the first year (<1 year) after screening, breast cancer risk was lowest among women without GA and with lower breast density (aHRs, 0.29, 0.52, and 0.79 for BI-RADS categories 1–3, respectively) and highest among women with BI-RADS category 4 and GA (aHR, 1.49; 95 % CI, 1.18–1.89). Notably, even in low-density breasts, the presence of GA was associated with short-term hazards at or above unity relative to the dense, no-GA reference (BI-RADS categories 1–3 with GA: aHRs, 1.25, 1.32, and 1.26, respectively). At the 1–2-year interval, the protective gradient associated with lower density persisted among women without GA (aHRs, 0.36, 0.58, and 0.84 for BI-RADS categories 1–3, respectively), whereas GA-related differences were attenuated and approached null values across densities (BI-RADS categories 1–4 with GA: aHRs, 1.20, 1.04, 0.97, and 1.03, respectively). Beyond two years of follow-up (≥2 years), aHRs remained below 1 across all non-reference density categories, regardless of GA status, ranging from 0.43 to 0.89. The association between GA and breast cancer risk varied according to BI-RADS density category (interaction P < .001 for GA × BI-RADS density).

**Table 4:** Breast cancer risk associated with global asymmetry by BI-RADS density category and interval since screening.

Interval since screening (years)	BI-RADS density category							
	BI-RADS 1		BI-RADS 2		BI-RADS 3		BI-RADS 4	
<1 year	**aHR**	**(95 % CI)**	**aHR**	**(95 % CI)**	**aHR**	**(95 % CI)**	**aHR**	**(95 % CI)**
No Global asymmetry	0.29	(0.25–0.32)	0.52	(0.47–0.57)	0.79	(0.72–0.85)	Ref.	
Global asymmetry	1.25	(0.88–1.77)	1.32	(1.08–1.63)	1.26	(1.06–1.50)	1.49	(1.18–1.89)
1–2 years								
No Global asymmetry	0.36	(0.33–0.40)	0.58	(0.53–0.63)	0.84	(0.79–0.90)	Ref.	
Global asymmetry	1.20	(0.89–1.62)	1.04	(0.85–1.26)	0.97	(0.83–1.15)	1.03	(0.81–1.31)
≥2 years								
No Global asymmetry	0.43	(0.42–0.44)	0.61	(0.60–0.63)	0.83	(0.81–0.84)	Ref.	
Global asymmetry	0.79	(0.70–0.90)	0.74	(0.68–0.79)	0.85	(0.80–0.89)	0.89	(0.82–0.96)

Abbreviations: aHR, adjusted hazard ratio; CI, confidence interval; BI-RADS, Breast Imaging Reporting and Data System; Ref, reference.

All aHRs were adjusted for age, body mass index, age at menarche, menopausal status, age at menopause, parity, breastfeeding history, oral contraceptive use, hormone replacement therapy, alcohol consumption, smoking status, physical activity, and family history of breast cancer.

The interaction P value for the association between BI-RADS density and GA were each <0.001.

## 4 Discussion

In this large nationwide screening cohort of over five million women, GA on mammography was a relatively uncommon finding (∼4 % of screening) and was more frequently observed in younger, premenopausal women with dense breasts. We found that the presence of GA was associated with a modest but statistically significant increase in breast cancer incidence during follow-up. Notably, this increase in risk was time-dependent: within the first year after the screening exam, women with GA had nearly twice the risk of being diagnosed with breast cancer compared to women without asymmetry, whereas beyond two years, this risk difference was minimal. The influence of breast density was also striking – in BI-RADS 1, GA conferred more than a twofold increase in breast cancer risk relative to women without GA, whereas among women with BI-RADS 4, the presence of asymmetry did not significantly elevate risk overall. These findings suggest that a unilateral GA may serve as an early radiologic indicator of an underlying malignancy in women with less dense breasts, potentially reflecting a mammographically occult cancer that becomes manifest shortly after GA is noted. In women with very dense breasts, however, GA appears to confer little additional predictive value.

The observation that GA is more commonly seen in younger, premenopausal women with dense breasts aligns with underlying physiologic and radiologic factors. Younger women typically have a higher proportion of dense breast tissue, whereas with increasing age and menopause, glandular tissue undergoes involution and is replaced by fat [14]. This dense tissue composition in younger women can develop asymmetrically – one breast may normally have more lobules or stromal tissue than the other – due to normal developmental variation or asymmetric hormonal stimulation [15]. Radiologically, dense fibroglandular tissue appears radiopaque on mammography, providing high contrast against fatty areas and making any imbalance between breasts more conspicuous on the image. In contrast, older women's breasts, which are more fatty, tend to have a more symmetric and homogeneous appearance on mammography [14], so a pronounced asymmetry is less common with age.

Our study demonstrated that women with GA had nearly a 90 % higher hazard of breast cancer in the first year after the screening, but beyond 2 years this risk had essentially no difference. GA seems to be a marker of near-term risk. This interpretation is consistent with findings from a previous study, which reported that marked volume asymmetry in breast density between sides was associated with cancers detected at the same screening visit and with interval cancers, but not with cancers found at the next routine screening visit [16]**.** GA could be more a harbinger of an existing cancer (one that will surface in the short term) rather than a general long-term risk factor like a genetic predisposition or consistently high breast density. Interestingly, Hall et al. incorporated bilateral differences (including density asymmetry or asymmetric lesions) into a model to identify women at high 2-year breast cancer risk, precisely to capitalize on this near-term predictive value [17].

Another important finding was the interaction between GA and breast density. GA conferred the greatest relative increase in risk among women with non-dense breasts, with progressively less impact as breast density increased. This density-dependent gradient in the effect of GA is both biologically and clinically intriguing. It suggests that when the background parenchyma is mostly fatty, any area of asymmetrically increased fibroglandular tissue is more likely to represent a significant abnormality or a localized predisposition, whereas in very dense breasts, small GAs may be less discernible (potentially under-reported). Prior research had hinted that asymmetry might predict risk independently of overall breast density [7], but to our knowledge no study had directly stratified the GA–cancer association by BI-RADS density. Our results reveal that the significance of GA as a risk marker is highly context-specific. GA in a predominantly fatty breast may be a red flag for underlying pathology, whereas in an extremely dense breast it appears to add little or no risk information beyond the already high baseline risk conferred by dense tissue. Several factors might explain this interaction. First, dense breasts already carry a roughly 4–6 fold higher risk of cancer relative to fatty breasts [6], so any additional risk from GA in that setting is marginal and harder to detect. Second, dense tissue can mask subtle asymmetric areas or make bilateral differences harder to appreciate on mammography, so some asymmetries in dense breasts might go unreported (a misclassification that would dilute the observed effect of GA in that group) [18,19]. In contrast, in a mostly fatty breast, even a modest unilateral increase in fibroglandular tissue stands out clearly against the fat background and could represent a real localized proliferation or evolving lesion that didn't meet criteria for immediate recall [18,19].

Our study has several limitations. First, GA was assessed based on radiologists’ screening reports, which introduces some subjectivity. We relied on whether “GA” was noted by the interpreting radiologist Misclassification of GA is possible [20]. However, this bias estimates toward the null in those dense categories [21]. Second, we could not distinguish new GA in this cohort. A “developing asymmetry” – meaning a new increase in density compared to prior imaging – is known to be a more ominous finding with a higher likelihood of malignancy [19]. Future analyses focusing on women with multiple sequential mammograms could investigate how changes in asymmetry over time relate to cancer risk. Third, while our cohort is extraordinarily large, it is observational. Residual confounding by factors we did not measure may have influenced the results. Nonetheless, prior studies have found that asymmetry remains a significant risk predictor even after accounting for many known risk factors [7], and our large sample size allowed us to adjust for a broad range of covariates. Finally, our cohort was drawn from a national screening program; thus, our findings may not fully generalize to populations that are not undergoing regular screening or to much younger women (<40, who are not included in our study). Despite these limitations, this study has several notable strengths. To our knowledge this study is the largest analysis to date of mammographic global asymmetry and subsequent breast cancer risk, and it provides new insights into how breast density and timing since screening modify the risk associated with this finding.

In conclusion, a GA on a screening mammogram show the breast cancer risk in the short term and is most pronounced in women with low breast density. From a clinical standpoint, while a GA is usually not considered an overtly suspicious finding by itself, our data suggest that its presence should heighten clinical vigilance — especially in women who otherwise have predominantly fatty breasts. Further research is warranted to understand the biological underpinnings of why GA might be linked to malignancy. Additionally, as personalized screening strategies are increasingly advocated, features like GA could help identify women who might benefit from adjunct imaging (such as ultrasound or MRI) or a shorter-interval follow-up after a “negative” mammogram.

## CRediT authorship contribution statement

**Sangjun Lee:** Writing – review & editing, Writing – original draft, Project administration, Conceptualization. **Soyeoun Kim:** Writing – review & editing, Writing – original draft, Visualization, Supervision, Resources, Project administration, Methodology, Formal analysis, Data curation, Conceptualization.

## Ethical approval

This retrospective nationwide cohort study was approved by the Institutional Review Board of Seoul National University Hospital (E−2406-021-1540) with a waiver of informed consent.

## Funding/support

This research did not receive any specific grant from funding agencies in the public, commercial, or not-for-profit sectors.

## Declaration of competing interest

The authors declare that they have no known competing financial interests or personal relationships that could have appeared to influence the work reported in this paper.

## Data Availability

Data analyzed in this study were obtained from the Korean National Health Insurance Service (NHIS) and are not publicly available. Requests for access to the data should be directed to the NHIS, subject to their data use policies.
